# Benchmarking the nutrient composition and labelling practices of dry or instant cereals for older infants and young children across seven Southeast Asian countries

**DOI:** 10.1111/mcn.13603

**Published:** 2023-12-13

**Authors:** Eleonora Bassetti, Jessica Blankenship, Jessica M. White, Lara Sweet, Diane Threapleton, Alissa M. Pries

**Affiliations:** ^1^ Helen Keller International, New York New York USA; ^2^ UNICEF East Asia Pacific Regional Office Bangkok Thailand; ^3^ JB Consultancy Johannesburg South Africa; ^4^ University of Leeds Leeds UK

**Keywords:** child nutrition, complementary feeding, complementary foods, food policy, infant and young child feeding, International Child Health Nutrition

## Abstract

In Southeast Asia, the increasing availability of commercially produced complementary foods (CPCF), including dry or instant cereals (CPCF cereals), has been noted, however, concerns exist around their nutrient profile and labelling practices. This 2021 study assessed the nutrient composition, labelling practices, and micronutrient content of CPCF cereals sold in the capital cities of seven Southeast Asian countries: Phnom Penh (Cambodia), Jakarta (Indonesia), Manila (Philippines), Bangkok (Thailand), Vientiane (Lao PDR), Hanoi (Vietnam), and Kuala Lumpur (Malaysia). The study adapted a nutrient profiling model from the WHO Regional Office for Europe to determine the proportion of products suitable for promotion for older infants and young children. Micronutrient content of fortified CPCF cereals was assessed against fortification levels specified in the Codex Alimentarius guideline for formulated complementary foods. Of the 484 products assessed, 184 (38.0%) met all nutrient composition requirements. Around one‐third of CPCF cereals contained added sugars and/or sweeteners (37.2%) and high levels of sodium (28.9%). None of the CPCF cereals met all labelling requirements, primarily due to the presence of inappropriate claims on the labels. Most fortified CPCF cereals contained adequate amounts of critical micronutrients, such as calcium, iron, zinc, vitamin A, and vitamin D. However, rates of fortification varied across the seven countries, and almost a third (30.8%) of CPCF cereals were not fortified with any micronutrients. To support the appropriate promotion of CPCF in the region, Southeast Asian countries need to strengthen and enforce national binding legal measures, including national standards for the composition, labelling, and fortification of CPCF cereals.

## Key messages

- Despite most commercially produced complementary foods (CPCF) cereals available in Southeast Asia met the majority of nutrient composition requirements, a considerable number of products contained added sugars/sweeteners (37.2%) and high levels of sodium (28.9%). Regulations that prohibit the use of added sugars/sweeteners and establish an upper limit for sodium in CPCF cereals are a policy priority in the region.
- None of the 484 CPCF cereals studied met all labelling requirements. Inappropriate claims were the most common. Three‐quarters of the products failed to meet labelling requirements on the protection and promotion of breastfeeding. National regulations on labelling must be strengthened and enforced to protect against inappropriate marketing of CPCF.
- Approximately two‐thirds or more of fortified CPCF cereals contained adequate amounts of micronutrients critical for older infants and young children (IYC), such as calcium, iron, zinc, vitamin A and vitamin D. Nonetheless, the fortification of CPCF cereals varied greatly by country, and almost a third (30.8%) of CPCF cereals were not fortified with any micronutrients. This is a missed opportunity to provide essential micronutrients to older IYC in contexts where nutrient‐poor diets are common. Future policies that stipulate mandatory minimum micronutrient levels for CPCF cereals are recommended for the region.

## Graphical abstract

This study assessed the nutrient composition, labelling practices, and micronutrient content of commercially produced complementary food (CPCF) cereals sold in seven Southeast Asian countries. Of the 484 products assessed, only one‐third met all nutrient composition requirements, however, most fortified CPCF cereals contained adequate amounts of critical micronutrients. Southeast Asian countries need to strengthen and enforce national binding legal measures, including national standards for the composition, labelling and fortification of CPCF cereals.

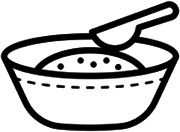

## 1 INTRODUCTION

During the complementary feeding period, from 6 to 23 months of age, children need adequate quantities of safe, nutrient‐dense foods in addition to breastmilk (WHO, 2003). Adherence to recommended feeding practices contributes to child health, growth and development, and reduce the risk of morbidity and obesity later in life (Black et al., 2013; De Onis & Branca, 2016; Lobstein et al., 2015). In Southeast Asia (SEA), while stunting and wasting persist as concerns, the rising prevalence of overweight children under 5 years indicates an emerging health challenge (UNICEF, WHO, et al., 2023). Micronutrient deficiencies are common among children 6–23 months of age, with anaemia being a severe public health problem in Cambodia and Lao People's Democratic Republic (PDR) (ASEAN UNICEF, & Alive&Thrive, 2022; WPF, 2022). In this context of a triple burden of malnutrition, prioritising nutrient‐dense complementary foods is essential to provide older infants and young children (IYC) critical nutrients for growth, development and long‐term health, as well as to establish healthy eating habits.

Consumption of commercially produced complementary foods (CPCF) is increasing in SEA due to the growing demand for convenience, limited caregiver time for meal preparation, and widespread promotion by CPCF manufacturers (UNICEF, 2020; Walls et al., 2023; Zehner et al., 2019). Of the various types of CPCF, dry or instant cereal (hereafter referred to as CPCF cereals) are among the first foods to be introduced to infants worldwide (Nicklas et al., 2020; Theurich et al., 2020). CPCF cereals are typically ground cereal‐based blends designed to be reconstituted with liquids. A recent survey targeting high socioeconomic status groups in Thailand, Malaysia, Indonesia and the Philippines found that over 90% of mothers of children 6–24 months regularly purchase CPCF cereals for feeding their child, indicating their importance as an energy source (Walls et al., 2023). When appropriately fortified, CPCF cereals can provide essential micronutrients often limited in the diets of older IYC (ASEAN UNICEF, & WFP, 2022; Csölle et al., 2022; Swanepoel et al., 2019; UNICEF, 2020; Zehner et al., 2019). However, some products may be of concern due to high sodium and/or sugar content (Bassetti et al., 2022; Bridge et al., 2021; De Araújo et al., 2023; Maalouf et al., 2017; Maslin & Venter, 2017; Scully et al., 2023). In addition to providing the highest nutrition quality, products marketed for older IYC must provide unambiguous information on their labels that will neither mislead consumers nor undermine public health recommendations (WHO Regional Office for Europe, 2022).

In 2016, the World Health Assembly (WHA) welcomed the World Health Organisation (WHO) Guidance on Ending the Inappropriate Promotion of Foods for Infants and Young Children (WHO Guidance) as part of the WHA resolution 69.9 (WHO, 2016). The WHO Guidance called for restrictions on inappropriate CPCF promotion so that they do not interfere with breastfeeding, contribute to obesity and non‐communicable diseases, create a dependency on commercial products or otherwise mislead caregivers, while ensuring that products have high nutritional quality (WHO, 2016). The WHO Guidance further encouraged the use of nutrient profile models (NPM) to determine CPCF suitability for children aged 6–36 months (WHO, 2016). To support the implementation of WHA 69.9, the WHO Regional Office for Europe created an NPM (WHO Europe NPM) to classify CPCF, including CPCF cereals, according to their nutrient composition and labelling practices (WHO Regional Office for Europe, 2019, 2022). The use of the WHO Europe NPM in the European region has provided evidence that many CPCF have inappropriate nutrient composition and labelling practices and are, therefore, not suitable for promotion for older IYC (Grammatikaki et al., 2021; Hutchinson et al., 2021; Santos et al., 2022; WHO Regional Office for Europe, 2019).

At present, in SEA, there is a lack of guidance at the national and regional level of what constitutes appropriate nutrient composition and package labelling for CPCF cereals, and limited evidence is available on the current landscape of CPCF cereals sold in the region (Blankenship et al., 2023). This study aimed to comprehensively assess the nutrient composition and labelling practices of CPCF cereals sold in seven SEA capital cities by analysing information from product labels. The study objectives included: (1) determining the proportion of CPCF cereals with suitable nutrient composition and labelling practices; (2) identifying the proportion of CPCF cereals that would require a ‘high sugar’ warning; and (3) assessing micronutrient contents reported on labels of fortified CPCF cereals against recommended nutrient intakes for older IYC.

## 2 METHODS

### 2.1 Study design

The study assessed CPCF cereals purchased between August and December 2021 in the capital cities of seven SEA countries: Cambodia, Indonesia, Lao PDR, Malaysia, Philippines, Thailand, and Vietnam. Capital cities were chosen because of their expected highest availability of CPCF, as they are among the largest cities of the country, and because they house UNICEF national offices where national researchers could be engaged. Within the Southeast Asian region, Singapore was excluded due to the absence of UNICEF operations in the country, and Myanmar due to its ongoing national emergency.

Adopting the WHO definition, CPCF products were identified as those explicitly marketed for older IYC, meeting any of the following criteria: (1) recommended for introduction at an age of less than 3 years; (2) labelled with the words “baby”, “infant”, “toddler”, ‘young child’ or a synonym; (3) labelled with an image of a child who appeared to be younger than 3 years of age or who was feeding with a bottle; or (4) in any other way presented as being suitable for children under 3 years of age (WHO, 2017). Labels with bottle images or preparation instructions suggesting bottle use were considered as “other ways” a product may present itself as suitable for children under the age of 3 years. Among the various categories of CPCF identified, this study specifically focused on CPCF cereals. CPCF cereals were defined as “dry rice, cereal, pulverised rusks or starchy root (at least 25% cereal and/or starch root content) with or without naturally sweet foods (such as dry fruit and powdered fruit juice), milk powder or whey powder,” to be prepared with the addition of liquids (WHO Regional Office for Europe, 2019). Findings relative to the other CPCF categories identified across the seven locations, such as purees, meals, snacks and finger foods, can be found in two associated papers (Bassetti et al., 2023; Pries et al., 2023).

Using information available on the labels, the nutrient composition and labelling practices of CPCF cereals were evaluated against relevant requirements by benchmarking them against an adapted version of the WHO Europe NPM (adapted NPM for CPCF) (WHO Regional Office for Europe, 2019). Adaptations included updates aligned with a newer version of the NPM that was being finalised at the time of the study (WHO Regional Office for Europe, 2022), and clearer definitions of claims made for CPCF (described in Supporting Information: Table S1). Additionally, the micronutrient content of fortified CPCF cereals was assessed against fortification levels specified in the Codex Alimentarius guideline for formulated complementary foods (Codex Alimentarius, 2013).

### 2.2 Store sampling and product identification

In Phnom Penh, Jakarta, Vientiane, Kuala Lumpur, Bangkok, and Hanoi, retail outlets selling CPCF were identified via two methods: a web‐based search (using the following terms: “supermarket (name of country)”, “grocery store (name of country)”, “department stores (name of country)”, “pharmacies (name of country)”, “baby store (name of country)”) and through consultation with local experts (e.g., Department of Trade and Industry; country industry associations representing retailers such as a Consumer Goods Council; health workers or nongovernment organisations involved in IYC nutrition). From this exercise, a list of chain and independent larger outlets including supermarkets, hypermarkets, large grocery stores, stand‐alone baby stores and large pharmacies, was developed for each country. For each identified major retail outlet, a web search was conducted to determine if they offered online or physical‐only retail. Online retailers without physical stores were excluded from this study. Due to the large number of retail outlets (*n* = 67) identified in Kuala Lumpur (Malaysia), approximately half (*n* = 21) of all chain stores per store category and nine independent stores were purposively sampled (prioritising stores with the greatest variety of CPCF).

The store selection was adapted for each country in response to the COVID‐19 pandemic. Due to safety concerns, visits were limited to online stores in Bangkok (*n* = 31), Jakarta (*n* = 25), Kuala Lumpur (*n* = 30), and Hanoi (*n* = 21). In these locations, retail outlets that did not have an online store was excluded from the study. In Phnom Penh, both physical (*n* = 22) and online stores (*n* = 6) were visited. In Vientiane, only physical stores (*n* = 22) were visited due to the low incidence of COVID‐19 and the scarcity of online shopping. All national researchers responsible for product acquisition and data extraction received standardised training, remotely administered by a dedicated technical team across all study sites. This training encompassed CPCF identification, categorisation, methodologies for sourcing CPCF in physical and online stores, and a detailed walkthrough on utilising the ONA Data app (https://ona.io/home/products/ona-data/features/) for data collection. The ONA Data app served as a centralised platform, ensuring consistent and efficient data collection and analysis throughout the research process.

For every physical store and online platform, the inventory was assessed for CPCF, and each unique CPCF purchased. Products were considered unique if they differed by brand name, sub‐brand name, descriptive name, age category/recommendation, manufacturer and/or flavour. Single‐serving and multi‐serving packages, different sizes of multi‐serving packages, and bundles of single‐serving sachets/packages of the same product were considered a single product, as were products that differed only by the type of packaging (e.g., box and canister). The ONA Data app was utilised to register products purchased both online and in physical stores, and subsequently it was used for all data capturing of label information. For online outlets, IYC‐related sections (such as “baby and kids” or “mother and baby”) and the refrigerated/frozen food sections were reviewed for CPCF products. When a search bar was present, local terms like “baby food”, “kid food”, or “toddler food” were used to ensure no CPCF was missed. In Phnom Penh and Vientiane, each physical store, including those with an online presence, was reviewed for CPCF. The search began in the “baby and toddler” section and extended to all sections of the store. If a store had both an online and a physical store, only CPCF not found online were purchased in the physical store. Due to the large number of CPCF products identified in Vietnam, only one of each unique product was purchased from all retail outlets in the retail outlet sample.

In Metropolitan Manila, the list of CPCF products was obtained from the Access to Nutrition Initiative CPCF analysis study conducted in 2020 (Access To Nutrition Initiative ATNI, 2021), and store sampling methodology has been previously described (Bassetti et al., 2022). The ATNI CPCF list was used to guide the online CPCF purchasing process, with national researchers using the list to search for products, verifying that the products found online matched the information provided across the company, brand, and product name. Any products no longer available online were excluded from the study.

### 2.3 Data capture and management

Once product purchasing was completed, CPCF duplicate products were deleted. Products were excluded if they did not provide label information in either English or the official language of the country. The labels of all unique products were photographed, and the images uploaded to the ONA Data app. Data extraction of label information using ONA was carried out independently by two national researchers in two steps. First, product names, ingredients and preparation instructions were reviewed to categorise products into one of the categories of the adapted NPM for CPCF. In the second step, nutrient composition and labelling practices information were extracted. For nutrient composition, the declaration of nutrition information per 100 g of the product as sold, serving size and ingredient list were extracted. For labelling practices, a measurable checklist of criteria was developed (Supporting Information: Table S1). Only label text in English or the local language was extracted, with local language taking precedence when both were present. When information was available in the local language only, this text was translated to English. Extracted data in ONA Data was exported into Microsoft Excel. A comparison of the double data entry was conducted, and all inconsistencies (e.g. data entry typos, or translation disagreement) reviewed and corrected. A final 5% error check was conducted and repeated until the error rate was below 5%.

### 2.4 Nutrient and labelling profiling methods

Using the extracted label information, products were benchmarked against the adapted NPM for CPCF to determine adherence to nutrient composition and labelling requirements. A CPCF cereal was categorised as suitable if it passed all nutrient composition and labelling requirements.

#### 2.4.1 Nutrient composition requirements

The adapted NPM for CPCF presents five nutrient composition requirements applicable to the CPCF cereals category: (1) no added sugar/sweeteners (including all mono‐ and disaccharides, syrups, nectars, honey, fruit juices or concentrated/powdered fruit juice (excluding lemon or lime juice), and nonsugar sweeteners); (2) dried/powdered fruit content <10% by weight; (3) total fat content ≤3.3 g/100 kcal (if the product does not contain added milk) and ≤4.5 g/100 kcal (if the product contains added milk); (4) protein content <5.5 g/100 kcal (if the product contains added milk); and (5) sodium content <50 mg/100 kcal as eaten. The presence of added sugars/sweeteners and the percentage of fruit content was determined based on ingredient lists. Where salt content was declared instead of sodium, sodium content was calculated based on the following: total sodium content = salt content/2.5. Products passed the nutrient composition assessment if they met all five requirements. If product labels were missing information for one of the nutrient composition requirements, the product was considered to have failed that requirement. The adapted NPM for CPCF, in line with the WHO Europe NPM, requires CPCF cereals to provide a front‐of‐pack ‘high sugar’ warning if the percentage energy from total sugar exceeds 30%. Products were assessed against this additional criterion, and results are presented separately to the adapted NPM for CPCF pass/fail assessment.

#### 2.4.2 Labelling requirements

Within the adapted NPM for CPCF, there are 12 labelling requirements applicable to the CPCF cereals category. These requirements broadly pertain to the protection and promotion of breastfeeding, claims, and product name and ingredient list clarity. A product passed the adapted NPM for CPCF labelling assessment if it met all 12 labelling requirements. If a product was missing information for one of the applicable labelling practices components, it failed that labelling requirement. Claims were categorised according to five categories: non‐permitted compositional claims, nutrient content claims, nutrient function claims, disease risk reduction claims, and other claims.

### 2.5 Micronutrient content assessment methods

Considering the high prevalence of micronutrient deficiencies among older IYC in SEA, a comprehensive analysis of the micronutrient content of CPCF cereals sold in the region was conducted in addition of the nutrient composition assessment based on the adapted NPM for CPCF. Products were categorised as fortified if ingredient lists included a fortificant (see Supporting Information: Table 2). The declared micronutrient contents of fortified CPCF cereals were assessed against the Codex “Guidelines for formulated complementary foods for older infants and young children” (Codex Alimentarius, 2013). Codex guidelines specify that a daily ration (defined as two servings) of a product should provide at least 50% of the reference nutrient intake from the Individual Nutrient Level 98 (INL_98_) (Codex Alimentarius, 2013). INL_98_ is the daily intake reference value that is estimated to meet the nutrient requirement of 98% of healthy individuals in a specific population (Lewis, 2019). Products were considered to meet the fortification guidelines if a daily ration of the product (defined as two servings) had a declared micronutrient quantity that was greater than or equal to the 50% reference intakes. The proportion of products meeting these guidelines was calculated for each micronutrient.

Values and units from the nutrient declaration on product labels were extracted for the following micronutrients: calcium, iron, zinc, copper, iodine, vitamin A, vitamin D, vitamin E, vitamin C, vitamin B12, vitamin B1, vitamin B2, vitamin B3, vitamin B6, folic acid, and vitamin K. For fortified CPCF cereals that provided nutrient content as a percentage of Recommended Dietary Allowance (% RDA) without also providing the nutrient content by weight, the latter was calculated using a method based on a 2019 study by Dreyfuss et al. (Dreyfuss et al., 2019). Specifically, the reported % RDA in one recommended serving (as listed on the product label) was multiplied by the RDA for the product's recommended age of use. When the product's recommended age of use spanned more than one age category for the specified country's RDA (e.g., 6–24 months), the average of the RDA values from the two age categories was used to calculate the nutrient content.

Quantities of each nutrient per 100 g of product and per serving were calculated based on serving size/net weight information provided on the product label. In cases where multiple serving sizes for children of different ages were provided on the product, the serving size corresponding to the lowest age range was used. Products missing serving size (*n* = 1) or nutrient information (*n* = 5) were excluded from the micronutrient content analysis.

### 2.6 Statistical analysis

The analysis was conducted with Stata (version 14.2). Descriptive statistics were calculated and summarised using proportions and frequencies.

## 3 RESULTS

### 3.1 Product characteristics

Across all countries, a total of 1881 products were identified, however, 246 were excluded as the language on the label was not in the official language of the country or English. Of the 1635 CPCF products included in this study, 484 (29.5%) were CPCF cereals. CPCF cereals accounted for 20.3% (*n* = 46) of total CPCF products in Phnom Penh (Cambodia), 41.2% (*n* = 112) in Jakarta (Indonesia), 23.7% (*n* = 28) in Vientiane (Lao PDR), 34.0%(*n* = 132) in Kuala Lumpur (Malaysia), 19.2% (*n* = 35) in Metropolitan Manila (Philippines), 16.5% (*n* = 34) in Bangkok (Thailand), and 40.0% (*n* = 97) in Hanoi (Vietnam). The final counts of CPCF cereals identified in each country are presented in Table 1. In Lao PDR and Cambodia, all CPCF cereals were imported. In Thailand (79.4%, *n* = 27) and Indonesia (72.3%, *n* = 81) the majority were locally produced, while less than half of the products in Malaysia (48.5%, *n* = 64), Vietnam (28.9%, *n* = 28), and the Philippines (20.0%, *n* = 7) were locally produced.

**Table 1:** Proportion of CPCF cereals passing relevant CPCF NPLM nutrient composition requirements of the adapted NPM for CPCF (*n* = 484).^a^

Country	*n*	No added sugar/sweetener^b^	Low/no added fruit^c^	Met sodium requirement^d^	Met protein requirement^e^	Met total fat requirement^f^	Met all nutrient requirements	Median # of nutrient requirements met
Cambodia	46	63.0 (29)	65.2 (30)	91.3 (42)	100.0 (46)	100.0 (46)	34.8 (16)	4 (3–5)
Indonesia	112	35.7 (40)	94.6 (106)	50.9 (57)	100.0 (112)	98.2 (110)	22.3 (25)	4 (2–5)
Lao PDR	28	71.4 (20)	57.1 (16)	82.1 (23)	100.0 (28)	100.0 (28)	25.0 (7)	4 (3–5)
Malaysia	132	85.6 (113)	81.8 (108)	71.2 (94)	100.0 (132)	100.0 (132)	56.1 (74)	5 (2–5)
Philippines	35	65.7 (23)	68.6 (24)	91.4 (32)	100.0 (35)	100.0 (35)	40.0 (14)	4 (3–5)
Thailand	34	79.4 (27)	58.8 (20)	82.4 (28)	100.0 (34)	100.0 (34)	44.1 (15)	4 (2–5)
Vietnam	97	53.6 (52)	89.7 (87)	70.1 (68)	100.0 (97)	99.0 (96)	34.0 (33)	4 (3–5)
All products	484	62.8 (304)	80.8 (391)	71.1 (344)	100.0 (484)	99.4 (481)	38.0 (184)	4 (2–5)

Abbreviations: CPCF, commercially produced complementary foods; NPM, nutrient profile models.

^a^ Values are presented as % (*n*) and median (range).

^b^ The following were considered added sugar/sweetener: all mono‐ and disaccharides (including sugars derived from fruits, sugarcane, palms or root vegetables, etc.); all syrups, nectars and honey (including molasses, agave, maple, blossom nectar, malted barley syrup and brown rice syrup, etc.); fruit juices or concentrated/powdered fruit juice, excluding lemon or lime juice (e.g. pear juice, concentrated apple juice or powdered mango juice); and nonsugar sweeteners (such as saccharin, acesulfame, aspartame, sucralose or stevia, etc.).

^c^ Requirement definition: < 10% by weight dried/powdered fruit.

^d^ Requirement definition: sodium <50 mg/100 kcal.

^e^ Requirement definition: <5.5 g/100 kcal total protein (if contains added milk).

^f^ Requirement definition: total fat ≤ 3.3 g/100 kcal (if does not contain added milk); total fat ≤4.5 g/100 kcal (if contains added milk).

### 3.2 Nutrient composition assessment

Of the 484 CPCF cereals assessed, 38.0% (*n* = 184) met all relevant nutrient composition requirements (Table 1). However, most products met four out of five requirements. CPCF cereals performed most poorly against the no added sugar/sweetener requirement, with more than one‐third (37.2%, *n* = 180) of the products not meeting this requirement. The presence of added sugars or sweeteners was particularly prevalent among products in Indonesia and Vietnam, with almost two‐thirds (64.3%, *n* = 72) and one‐half (46.4%, *n* = 45) of products, respectively, listing a sugar or sweetening agent in their ingredient lists. The sodium requirement was met by just under three‐quarters (71.1%, *n* = 344) of CPCF cereals, including the majority of products in the Philippines, Cambodia, Lao PDR and Thailand. Whereas half of the products in Indonesia (49.1%, *n* = 55) and almost one‐third in Malaysia (28.8%, *n* = 38) and Vietnam (29.9%, *n* = 29) contained more than 50 mg of sodium per 100 kcal. The majority (80.8%, *n* = 391) of CPCF cereals met the low/no‐added fruit content requirement. Across the seven countries, all CPCF cereals met the protein requirement, and almost all (99.4%, *n* = 481) met the total fat requirement.

The adapted NPM for CPCF recommends CPCF cereals provide a front‐of‐pack “high sugar” warning if total sugar content exceeds 30% of total energy. Of the 339 (70.0%) products declaring total sugar content on their labels, only two (0.6%) CPCF cereals (both from Indonesia) contained total sugar content that would warrant a “high sugar” warning on their labels.

### 3.3 Labelling practices assessment

None of the 484 CPCF cereals passed the labelling practices assessment, primarily due to the presence of claims on labels (Table 2). Non‐permitted compositional claims and “other claims” were common across all seven countries, while disease risk reduction claims were rare. Nearly three‐quarters (72.3%, *n* = 350) of products displayed nutrient content claims on their labels, with this practice most common in Vietnam, Indonesia, and the Philippines. Nutrient function claims were particularly prevalent in Vietnam (60.7%, *n* = 17) and Lao PDR (60.8%, *n* = 59).

**Table 2:** Proportion of CPCF cereals passing relevant labelling requirements of the adapted NPM for CPCF (*n* = 484).^a^

	Cambodia (*n* = 46)	Indonesia (*n* = 112)	Lao PDR (*n* = 28)	Malaysia (*n* = 132)	Philippines (*n* = 35)	Thailand (*n* = 34)	Vietnam (*n* = 97)	All products (*n* = 484)
**Protection and promotion of breastfeeding**								
Minimum recommended age of introduction ≥6 months	65.2 (30)	92.0 (103)	92.9 (26)	94.7 (125)	80.0 (28)	94.1 (32)	85.6 (83)	88.2 (427)
Not marketed as suitable for <6 months	78.3 (36)	58.9 (66)	92.9 (26)	59.1 (78)	91.4 (32)	100.0 (34)	58.8 (57)	68.0 (329)
Message on importance of breastfeeding ≥2 years	23.9 (11)	41.1 (46)	7.1 (2)	57.6 (76)	11.4 (4)	0.0 (0)	0.0 (0)	28.7 (139)
Does not suggest superiority or equivalence to breastmilk	97.8 (45)	100.0 (112)	100.0 (28)	88.6 (117)	97.1 (34)	97.1 (33)	100.0 (97)	96.3 (466)
Does not recommend or promote bottle feeding	100.0 (46)	100.0 (112)	100.0 (28)	99.2 (131)	100.0 (35)	100.0 (34)	100.0 (97)	99.8 (483)
**Met all labelling requirements for protection and promotion of breastfeeding**	**21.7 (10)**	**30.4 (34)**	**7.1 (2)**	**37.9 (50)**	**11.4 (4)**	**0.0 (0)**	**0.0 (0)**	**20.7 (100)**
**Claims**								
No non‐permitted compositional claims	15.2 (7)	11.6 (13)	32.1 (9)	13.6 (18)	17.1 (6)	17.7 (6)	16.5 (16)	15.5 (75)
No nutrient content claims	41.3 (19)	17.9 (20)	35.7 (10)	39.4 (52)	17.1 (6)	35.3 (12)	15.5 (15)	27.7 (134)
No nutrient function claims	50.0 (23)	47.3 (53)	39.3 (11)	75.0 (99)	82.9 (29)	61.8 (21)	39.2 (38)	56.6 (274)
No disease risk reduction claims	100.0 (46)	99.1 (111)	100.0 (28)	97.0 (128)	100.0 (35)	100.0 (34)	94.9 (92)	97.9 (474)
No other claims	34.8 (16)	7.1 (8)	21.4 (6)	22.7 (30)	11.4 (4)	0.0 (0)	24.7 (24)	18.2 (88)
**Met all labelling requirements for claims**	**0.0 (0)**	**0.0 (0)**	**0.0 (0)**	**0.0 (0)**	**0.0 (0)**	**0.0 (0)**	**3.1 (3)**	**0.6 (3)**
**Product name and ingredient list clarity**								
Product name reflects ingredients in descending order as per ingredient list	80.4 (37)	72.3 (81)	92.9 (26)	74.2 (98)	54.3 (19)	41.2 (14)	48.5 (47)	66.5 (322)
Percentage of fruit stated in ingredient list^b^	50.0 (10)	95.0 (19)	100.0 (14)	42.9 (12)	56.3 (9)	100.0 (20)	72.7 (16)	71.4 (100)
**Met all labelling requirements for product name and ingredient list clarity**	**63.0 (29)**	**71.4 (80)**	**92.9 (26)**	**65.2 (86)**	**48.6 (17)**	**41.2 (14)**	**46.4 (45)**	**61.4 (297)**
**Met all relevant labelling requirements**	**0.0 (0)**	**0.0 (0)**	**0.0 (0)**	**0.0 (0)**	**0.0 (0)**	**0.0 (0)**	**0.0 (0)**	**0.0 (0)**

Abbreviations: CPCF, commercially produced complementary foods; NPM, nutrient profile models.

^a^ Values are presented as % (*n*).

^b^ Question applicable to 140 products containing fruit: Cambodia *n* = 20; Indonesia *n* = 20; Lao PDR *n* = 14; Malaysia *n* = 28; Philippines *n* = 16; Thailand *n* = 20; Vietnam *n* = 22.

There was mixed performance across the five labelling requirements intended to protect and promote breastfeeding. Less than one‐third (28.7%, *n* = 139) of CPCF cereals presented a message on the importance of continued breastfeeding to 2 years or beyond. Only two (7.1%) products in Lao PDR, four (11.4%) in the Philippines and none in Vietnam and Thailand met this requirement. The majority of CPCF cereals (88.2%, *n* = 427) presented a recommended age of introduction of at least 6 months on the label, however, more than one‐third (34.8%, *n* = 16) of products in Cambodia did not meet this requirement. Such products sold in Cambodia either provided no age recommendation (*n* = 7) or provided an age recommendation from 4 months (*n* = 9). Across all seven countries, nearly one‐third (32.0%, *n* = 155) of CPCF cereals were marketed as suitable for children under 6 months. Dry or instant cereals found in Indonesia, Malaysia, and Vietnam performed particularly poorly against this requirement, with almost half of the products using images or text that suggested suitability for infants under 6 months of age. Most products (96.3%, *n* = 466) in all seven countries did not suggest superiority or equivalence to breastmilk.

Almost two‐thirds (61.4%, *n* = 297) of CPCF cereals met both labelling requirements relative to the product name and ingredient list clarity. Two‐thirds (66.5%, *N* = 322) had names that reflected their ingredients in descending order, as per their ingredient lists. Across the seven countries, almost three‐quarters (71.4%, *n* = 100) of CPCF cereals that contained fruit stated the percentage of fruit in the ingredient list. While all fruit‐containing products in Lao PDR and Thailand, and almost all products in Indonesia (95.0%, *n* = 19) met this requirement, far fewer of the products in Malaysia (42.9%, *n* = 12), Cambodia (50.0%, *n* = 10) and the Philippines (56.3%, *n* = 9) stated the percentage of fruit in the ingredient list.

### 3.4 Micronutrient content assessment

Based on the analysis of labels across the seven countries, approximately two‐thirds of CPCF cereals were fortified with any micronutrient (69.2%, *n* = 335). The three most common fortificants were vitamin B1, iron, and calcium, present in approximately 65.7%, 60.1%, and 56.6% of the products, respectively (Table 3). Additionally, over half of products were fortified with zinc, vitamin A, vitamin D, vitamin E, and vitamin C. However, rates of fortification varied across countries. For instance, while 78.3% of CPCF cereals in Cambodia and 76.8% in Indonesia were fortified with iron, the rates were much lower in Malaysia (34.1%) and Thailand (29.4%). Most fortified CPCF cereals met Codex fortification levels for iron, zinc, calcium, vitamin A and vitamin D. Specifically, most CPCF cereals fortified with calcium met the Codex target for calcium per daily ration, but a lower proportion of products met this target in Cambodia (41.7%, *n* = 15), and Malaysia (50.0%, *n* = 17). The majority of iron‐fortified CPCF cereals met the Codex target for iron. However, over half of these products in the Philippines (51.7%, *n* = 15) failed to meet this target. Of the 250 zinc‐fortified products, 75.2% (*n* = 188) met the zinc target; a lower proportion met the target in Cambodia (58.3%, *n* = 21), Lao PDR (54.5%, *n* = 6) and Malaysia (51.6%, *n* = 16). Approximately 38.0% (*n* = 184) of CPCF cereals fortified with vitamin B12 met the vitamin B12 target, with variations by country ranging from 75.9% in Indonesia to only 10.6% in Malaysia. In all seven countries, most vitamin A‐fortified products met the vitamin A target, and most vitamin D‐fortified products met the vitamin D target. Median micronutrient content of CPCF cereals across each of the seven SEA countries is detailed in Supporting Information: Table S3.

**Table 3:** Proportion of fortified CPCF cereals meeting micronutrient levels specified in the Codex Alimentarius guideline for formulated complementary foods by daily ration.^a^

Country	Cambodia (*n* = 46)	Indonesia (*n* = 112)	Lao PDR (*n* = 28)	Malaysia (*n* = 132)	Philippines (*n* = 35)	Thailand (*n* = 34)	Vietnam (*n* = 97)	All products (*n* = 484)
Fortified with calcium	78.3 (36)	78.6 (88)	39.3 (11)	25.8 (34)	68.6 (24)	26.5 (9)	74.2 (72)	56.6 (274)
Calcium 50% INL_98_ b	41.7 (15)	65.9 (58)	63.6 (7)	50.0 (17)	70.8 (17)	88.9 (8)	66.7 (48)	62.0 (170)
Fortified with iron	78.3 (36)	76.8 (86)	46.4 (13)	34.1 (45)	82.9 (29)	29.4 (10)	74.2 (72)	60.1 (291)
Iron 50% INL_98_ b	80.6 (29)	100.0 (86)	84.6 (11)	75.6 (34)	48.3 (14)	90.0 (9)	88.9 (64)	84.9 (247)
Fortified with zinc	78.3 (36)	75.9 (85)	39.3 (11)	23.5 (31)	62.9 (22)	26.5 (9)	57.7 (56)	51.7 (250)
Zinc 50% INL_98_ b	58.3 (21)	89.4 (76)	54.5 (6)	51.6 (16)	77.3 (17)	88.9 (8)	78.6 (44)	75.2 (188)
Fortified with copper	0.0 (0)	0.0 (0)	0.0 (0)	0.0 (0)	0.0 (0)	0.0 (0)	9.3 (9)	1.9 (9)
Copper 50% INL_98_ b	0.0 (0)	0.0 (0)	0.0 (0)	0.0 (0)	0.0 (0)	0.0 (0)	0.0 (0)	0.0 (0)
Fortified with iodine	43.5 (20)	72.3 (81)	25.0 (7)	12.1 (16)	42.9 (15)	23.5 (8)	56.7 (55)	41.7 (202)
Iodine 50% INL_98_ b	30.4 (14)	57.1 (64)	14.3 (4)	7.6 (10)	25.7 (9)	8.8 (3)	33.0 (32)	28.1 (136)
Fortified with vitamin A	60.9 (28)	78.6 (88)	28.6 (8)	23.5 (31)	60.0 (21)	26.5 (9)	74.2 (72)	53.1 (257)
Vitamin A 50% INL_98_ b	71.4 (20)	80.7 (71)	87.5 (7)	71.0 (22)	81.0 (17)	88.9 (8)	77.8 (56)	78.2 (201)
Fortified with vitamin D	60.9 (28)	78.6 (88)	28.6 (8)	23.5 (31)	62.9 (22)	17.6 (6)	74.2 (72)	52.9 (256)
Vitamin D 50% INL_98_ b	71.4 (20)	97.7 (86)	87.5 (7)	80.6 (25)	77.3 (17)	100.0 (6)	94.4 (68)	89.8 (230)
Fortified with vitamin E	65.2 (30)	75.0 (84)	39.3 (11)	24.2 (32)	51.4 (18)	20.6 (7)	68.0 (66)	51.2 (248)
Vitamin E 50% INL_98_ b	39.1 (18)	60.7 (68)	25.0 (7)	17.4 (23)	48.6 (17)	20.6 (7)	64.9 (63)	41.9 (203)
Fortified with vitamin C	65.2 (30)	75.0 (84)	39.3 (11)	25.8 (34)	65.7 (23)	26.5 (9)	70.1 (68)	53.5 (259)
Vitamin C 50% INL_98_ b	65.2 (30)	75.0 (84)	39.3 (11)	19.7 (26)	45.7 (16)	23.5 (8)	62.9 (61)	48.8 (236)
Fortified with vitamin B12	65.2 (30)	76.8 (86)	39.3 (11)	22.7 (30)	34.3 (12)	20.6 (7)	55.7 (54)	47.5 (230)
Vitamin B12 50% INL_98_ b	32.6 (15)	75.9 (85)	25.0 (7)	10.6 (14)	31.4 (11)	20.6 (7)	46.4 (45)	38.0 (184)
Fortified with vitamin B1	84.8 (39)	78.6 (88)	50.0 (14)	40.2 (53)	82.9 (29)	32.4 (11)	86.6 (84)	65.7 (318)
Vitamin B1 50% INL_98_ b	65.2 (30)	50.0 (56)	35.7 (10)	28.8 (38)	62.9 (22)	32.4 (11)	83.5 (81)	51.2 (248)
Fortified with vitamin B2	58.7 (27)	74.1 (83)	39.3 (11)	23.5 (31)	51.4 (18)	26.5 (9)	55.7 (54)	48.1 (233)
Vitamin B2 50% INL_98_ b	41.3 (19)	59.8 (67)	25.0 (7)	170.5 (225)	48.6 (17)	26.5 (9)	44.3 (43)	38.6 (187)
Fortified with vitamin B3	65.2 (30)	75.0 (84)	42.9 (12)	25.0 (33)	57.1 (20)	8.8 (3)	51.5 (50)	47.9 (232)
Vitamin B3 50% INL_98_ b	43.5 (20)	58.0 (65)	25.0 (7)	18.2 (24)	48.6 (17)	8.8 (3)	48.5 (47)	37.8 (183)
Fortified with vitamin B6	65.2 (30)	72.3 (81)	39.3 (11)	23.5 (31)	51.4 (18)	20.6 (7)	55.7 (54)	47.9 (232)
Vitamin B6 50% INL_98_ b	39.1 (18)	60.7 (68)	25.0 (7)	18.9 (25)	48.6 (17)	20.6 (7)	52.6 (51)	39.9 (193)
Fortified with folic acid	58.7 (27)	75.0 (84)	39.3 (11)	22.7 (30)	48.6 (17)	8.8 (3)	55.7 (54)	46.7 (226)
Folic acid 50% INL_98_ b	2.2 (1)	0.0 (0)	0.0 (0)	0.0 (0)	0.0 (0)	0.0 (0)	14.4 (14)	3.1 (15)
Fortified with vitamin K	37.0 (17)	19.6 (22)	0.0 (0)	0.0 (0)	5.7 (2)	0.0 (0)	30.9 (30)	14.7 (71)
Vitamin K 50% INL_98_ b	23.9 (11)	18.8 (21)	0.0 (0)	0.0 (0)	5.7 (2)	0.0 (0)	26.8 (26)	12.4 (60)

Abbreviations: CPCF, commercially produced complementary foods; INL_98_, individual nutrient level 98; Lao PDR, Lao People's Democratic Republic.

^a^ Values are presented as % (*n*).

^b^ Denominators are the number of products fortified with this nutrient.

## 4 DISCUSSION

This study benchmarked CPCF cereals sold in seven SEA countries against an adapted nutrient profiling model for nutrient composition and labelling requirements and against Codex Alimentarius micronutrient content guidelines. Of the 484 products assessed, 38.0% (*n* = 184) met all nutrient composition requirements, and no products met all labelling requirements. Therefore, none of the CPCF cereals were deemed suitable for promotion for older IYC. The evaluation of micronutrient content of fortified CPCF cereals showed that most products were appropriately fortified, meeting the Codex specification of 50% INL_98_ per daily ration. However, rates of fortification varied across countries, indicating a missed opportunity for improving the consumption of essential micronutrients, particularly among older IYC in the region.

The presence of added sugars/sweeteners was the most common reason why CPCF cereals failed the nutrient composition assessment, an issue previously noted as a concern in studies conducted in Cambodia, Indonesia and the Philippines (Bassetti et al., 2022), Australia (Scully et al., 2023), and Europe (Garro‐Mellado et al., 2022; Hutchinson et al., 2021). Despite over one‐third of CPCF cereals containing added sugars/sweeteners, only two products contained total sugar warranting a “high sugar” warning on their labels. This finding is consistent with a study conducted in Europe, which found few CPCF cereals exceeding a threshold of 30% of energy from total sugar (Hutchinson et al., 2021). International authorities are unanimous in recommending that complementary foods should not be prepared with added sugars (Fidler Mis et al., 2017; WHO Regional Office for Europe, 2022), and a recent study among caregivers of older IYC in Southeast Asia found that the majority cited sugar content as one of the main concerns when purchasing CPCF (Walls et al., 2023). The total added and free sugar contents of CPCF cereals are of concern because sugar intake is linked to later health status, including development of dental caries and weight gain (Breda et al., 2019). Exposure to products with added sugars during infancy can promote a preference for sweet foods and poor eating habits in childhood (Foterek et al., 2016). Furthermore, it is critical to note that 30.0% (*n* = 145) of CPCF cereals did not declare total sugar content on their labels, and thus could not be assessed against the front‐of‐pack ‘high sugar’ warning. Additionally, approximately 5% of all products, primarily from Malaysia and Vietnam, did not declare sodium content and thus could not be assessed against the sodium requirement. A similar observation was made in a recent study in the Russian Federation (Kontsevaya et al., 2023). The lack of this information hinders well‐informed decision‐making for caregivers of older IYC. To address this issue, there is a need to require additional mandatory nutrient content information on the labels of CPCF cereals, including total sugar content.

To a degree, product nutritional performance within each country can be explained by existing legal requirements. For instance, Malaysia, Vietnam and Indonesia have regulations on the maximum fructose content in CPCF cereals, and in all three of these countries the vast majority of CPCF cereals met the fruit content requirement of 10% (dry weight). In comparison, a significant proportion of products in Cambodia (34.8%), Lao PDR (42.9%), the Philippines (31.4%), and Thailand (41.2%) exceeded the maximum amount of added fruit, all countries where there is no regulation on fructose content specifically, or sugar content overall. In Indonesia, Malaysia, and Vietnam, Codex standards concerning sugar content of CPCF cereals have been adopted: maximum amounts of added sucrose, fructose, glucose, glucose syrup, or honey are specified, but exceeding the maximum levels is not prohibited by law (Head of Drug and Food Supervisory Board BPOM, 2020; Ministry of Health of Malaysia, 1985; Ministry of Health of Vietnam, 2012). To ensure that older IYC is not exposed to unnecessarily sweet products, adopting binding legal measures that prohibit added sugars or sweeteners in CPCF should be high priority in governmental policy in SEA.

While in most of the study countries, the majority of CPCF cereals had sodium levels below the maximum sodium requirement, roughly half (49.1%, *n* = 55) of products available in Indonesia and one‐third in Malaysia (28.8%, *n* = 38), and Vietnam (29.9%, *n* = 29) exceeded this requirement. This finding is consistent with a previous study conducted in Indonesia which showed that almost two‐thirds (65.2%) of CPCF cereals exceeded the sodium requirement (Bassetti et al., 2022). While, studies conducted in other locations usually reported a low proportion of CPCF cereals presenting high sodium levels (De Araújo et al., 2023; Scully et al., 2023). According to Indonesian, Malaysian and Vietnamese regulations, sodium content for CPCF cereals shall not exceed 100 mg/100 kcal (Head of Drug and Food Supervisory Board BPOM, 2020; Ministry of Health of Malaysia, 1985; Ministry of Health of Vietnam, 2012), in alignment with Codex standard (Codex Alimentarius, 2019). However, this limit is twice as high as the adapted NPM for CPCF requirement, which suggest 50 mg/100 kcal to prevent children becoming accustomed to a diet with high salt content. High sodium intake in early life is correlated with high blood pressure in childhood and adulthood, meaning that children with increased blood pressure are at high risk for hypertension and its related morbidities as adults (Lava et al., 2015). Moreover, taste preferences are developed during infancy and childhood, therefore decreasing the exposure to salty‐tasting foods is recommended to ensure a decreased salt intake later in life (Lava et al., 2015; Liem, 2017). A strict upper limit for sodium that is in line with WHO recommendations should be included in national and regional regulations (WHO Regional Office for Europe, 2022). The large array of products already meeting the WHO sodium requirement demonstrates that this target is easily achievable within this category of CPCF cereals.

Inappropriate claims were the most common reason for CPCF cereals failing the labelling practices assessment of the adapted NPM for CPCF. Similarly, extensive use of claims has been noted on CPCF products in the UK (Garcia et al., 2022) and in Taiwan (Koo et al., 2018). Previous research has shown that CPCF cereals presenting nutritional and health claims commonly had a less desirable nutrient profile when compared to those without such claims (Bassetti et al., 2022; Koo et al., 2018). CPCF cereals that include claims are likely to attract caregivers who perceive products to be more nutritious for their children (Harris et al., 2011). Moreover, claims can create a healthy “halo effect”, establishing brand loyalty, idealising the product and implying superiority over other foods (Harris et al., 2011). Codex guidelines state that “nutrition and health claims shall not be permitted for foods for infants and young children except where specifically provided for in relevant Codex standards or national legislation” (FAO, & WHO, 1997), and the WHO Europe NPM states that no claims should be allowed on CPCF (WHO Regional Office for Europe, 2022). Despite regulations restricting claims on products for older IYC in Malaysia (Ministry of Health of Malaysia, 1985), Indonesia (Head of Drug and Food Supervisory Board BPOM, 2016), Lao PDR (Ministry of Health of Lao People's Democratic Republic, 2009), Philippines (Department of Health DoH of the Philippines, 2014) and Vietnam (Ministry of Health of Vietnam, 2014a), the use of claims on CPCF cereals is ubiquitous in these countries. Our findings indicate that further revision and stronger enforcement of national regulations and manufacturer compliance are urgently needed to protect older IYC diets from misleading marketing. Furthermore, regulations to prevent inappropriate claims on CPCF cereals should be introduced in Cambodia and Thailand, where binding legal measures are still absent.

Over three‐quarters of CPCF cereals included in this study failed to meet the five labelling requirements on the protection and promotion of breastfeeding. Despite national regulations (Head of Drug and Food Supervisory Board BPOM, 2020; Ministry of Health of Malaysia, 1985; Ministry of Health of Vietnam, 2014b), approximately forty percent of products available in Indonesia, Malaysia, and Vietnam presented images and/or text that suggested suitability for infants under 6 months of age. Over one‐third of products available in Cambodia did not provide any age recommendation on the label or provided an age recommendation from 4 months. Inappropriate product marketing before 6 months of age may undermine optimal feeding practices by encouraging early food introduction and displacing breastmilk intake (Smith et al., 2015). In SEA, breastfeeding practices are suboptimal, with less than half of infants under 6 months being exclusively breastfed in Malaysia, Vietnam, and Lao PDR, and only 14% in Thailand (UNICEF, 2023). Moreover, continued breastfeeding is very low, with less than a quarter of children still breastfed at 2 years of age in Thailand and Vietnam, and less than half in Lao PDR and Malaysia (UNICEF, 2023). The findings from this SEA study suggest that additional specificity is needed in national legislation to strengthen labelling regulations on the protection and promotion of breastfeeding, alongside enforcement of existing measures.

In all seven countries, most fortified CPCF cereals met the Codex target of 50% INL_98_ for calcium, iron, zinc, vitamin D and vitamin A. However, fortification status varied by country, with a noticeable proportion of products not being fortified. Older IYC are at a critical stage in their development and require adequate intake of micronutrients to support growth and development (Kathryn, 2001, 2013). In contexts where nutrient‐poor diets are prevalent, well‐fortified CPCF cereals can be an important tool to fill the micronutrient gap and prevent micronutrient deficiencies of children under 3 years of age (Csölle et al., 2022; Eichler et al., 2012; UNICEF, 2020; WHO, 2003). Failure to provide sufficient amounts of essential micronutrients can result in a higher risk of micronutrient deficiencies, which can have long‐term consequences on cognitive and physical development (De Onis & Branca, 2016; Kathryn, 2013; Lutter et al., 2021; UNICEF, 2020). In SEA, several studies based on linear programming demonstrated that it may be challenging to meet older IYC's nutritional needs, particularly for iron, zinc and calcium, without fortified foods (Chittchang et al., 2022; Dewey & Adu‐Afarwuah, 2008; Fahmida & Santika, 2016; Ferguson et al., 2019; Mejos et al., 2021). A study conducted in Indonesia showed that exclusion of fortified foods from the diets of children 12–23 months of age was associated with inadequate intake of more nutrients in the complementary feeding diet (Fahmida & Santika, 2016). Although Malaysia, Indonesia, Vietnam, and the Philippines have policies concerning fortification standards of CPCF cereals (Bureau of Food and Drugs BFAD of the Philippines, 1995; Head of Drug and Food Supervisory Board BPOM, 2020; Ministry of Health of Malaysia, 1985; Ministry of Health of Vietnam, 2014a), Cambodia, Lao PDR, and Thailand currently have no regulations in place. Our findings suggest that many manufacturers are missing the opportunity to improve child diets by fortifying CPCF cereals, which are similar to traditional rice/cereal porridges commonly fed to children in SEA, with micronutrients. We recommend that future national and regional regulations in SEA mandate minimum micronutrient levels for CPCF cereals to ensure that adequate amounts of micronutrients are present in these products to meet the requirements of older IYC.

Growing demand and supply of commercial CPCF cereals in SEA requires robust and enforceable regulations to control the nutrient composition, labelling practices and micronutrient content of these products. Codex standards provide global guidance for developing national regulations in each of these areas for CPCF cereals. However, recently published WHO Guidance recognises that current Codex standards are inadequate to determine the suitability of contemporary commercial products marketed for older IYC, and calls for new or updated Codex standards that are in full alignment with WHO recommendations (WHO, 2017). In SEA, current national regulations are heterogeneous: most of them only partially align with the Codex standards, and do not comply with WHO recommendations (Blankenship et al., 2023). To ensure nutritionally appropriate products for older IYC, it is crucial to enforce existing regulations, and develop or update national standards for CPCF cereals, that align with Codex standards and WHO recommendations for both macro and micronutrients.

The study presents some limitations. While we obtained data on 484 CPCF cereals across seven countries in SEA through primary data collection, it is important to note that product identification was limited to capital cities of each country. Therefore, the sample of CPCF cereals obtained may not be exhaustive of all products available in the market in these countries. Moreover, due to COVID‐19 outbreak, adjustments in store sampling methodology (physical vs. online) were made based on the specific context of each country. In Indonesia, Malaysia, Thailand, and Vietnam, only online platforms were searched for CPCF. Therefore, store categories that may not have had an online presence (e.g., pharmacies, independent supermarkets, and baby stores) were not included in the store sampling. Nevertheless, given that most large stores selling CPCF had an online store in these countries, this decision likely had a minimal impact on the findings.

## 5 CONCLUSION

This study reveals that a concerning number of CPCF cereals sold in SEA contained added sugars/sweeteners (37.2%) and/or high levels of sodium (28.9%). Most products presented inappropriate labelling practices, with the use of claims being the predominant inappropriate practice. Additionally, despite most fortified CPCF cereals containing adequate amounts of critical micronutrients, nearly one‐third of CPCF cereals were not fortified with any micronutrient. Appropriately fortified CPCF cereals, with optimal nutrient composition and labelling practices, represent a critical opportunity to fill existing nutrient gaps in older IYC's diets in SEA. Strong, unambiguous nationally binding legal measures, including national standards for composition and labelling of CPCF cereals, should be strengthened and enforced to ensure that caregivers have accurate information and are protected from inappropriate marketing of CPCF cereals and that older IYC are consuming nutritious CPCF.

## AUTHOR CONTRIBUTIONS

Jessica Blankenship and Alissa M. Pries designed the study. Lara Sweet and Alissa M. Pries conducted the data management. Alissa M. Pries, Jessica Blankenship, and Eleonora Bassetti analysed the data. Eleonora Bassetti wrote the paper, and all authors provided substantial reviews.

## CONFLICT OF INTEREST STATEMENT

The authors declare no conflict of interest.

## Supporting information

Supporting information.

Supporting information.

Supporting information.

## Data Availability

The data that support the findings of this study are available from the corresponding author upon reasonable request.
