# Supplementary material for: Benchmarking the nutrient composition and labelling practices of dry or instant cereals for older infants and young children across seven Southeast Asian countries
Source: Matern Child Nutr. 2023 Dec 13;19(Suppl 2):e13603. doi: 10.1111/mcn.13603 (PMC10719057; doi:10.1111/mcn.13603)
Supplement: Supplementary file 1 — Supporting information. [file MCN-19-e13603-s003.docx]

| Supplemental Table 1. Types and definitions of claims assessed for as part of the CPCF NPLM assessment | |
| --- | --- |
| Type of claim | Definition and examples |
| Non- permitted compositional claims | Text stating/ implying that the composition of the product is different/ special, that that an ingredient has not been added to a food or that compares the nutrient levels and/ or energy value of the product to other products and/or brands.  Example: “Natural ingredients”, “No added preservatives” |
| Nutrient content claims^1^ | A nutrition claim that describes the level of a nutrient contained in a food.  Example: “Source of calcium”, “9 Vitamins and Minerals” |
| Nutrient function claims^1^ | A nutrition claim that describes the physiological role of the nutrient in growth, development, and normal functions of the body.  Example: “Nutrient A (naming a physiological role of nutrient A in the body in the maintenance of health and  promotion of normal growth and development). Food X is a source of/ high in nutrient A.” |
| Reduction of disease risk claims^1^ | Claims relating the consumption of a food or food constituent, in the context of the total diet, to the reduced risk of developing a disease or health-related condition.  Risk reduction means significantly altering a major risk factor(s) for a disease or health-related condition. Diseases have multiple risk factors and altering one of these risk factors may or may not have a beneficial effect. The presentation of risk reduction claims must ensure, for example, by use of appropriate language and reference to other risk factors, that consumers do not interpret them as prevention claims.  Example: “A healthful diet low in nutrient or substance A may reduce the risk of disease D.  Food X is low in nutrient or substance A.” |
| Other claims | Includes all other claims made that are not related to compositional, nutrient content, nutrient function, or disease risk reduction. Includes marketing claims related to taste, quality and texture of the food, as well as convenience/lifestyle amongst others.  Examples: “Easy to swallow texture.”, “Great for a busy and active lifestyle.” |

^1^Definition based on Codex Alimentarius Guidelines for use of nutrition and health claims, CAC/GL 23 – 1997
