# Supplementary material for: Benchmarking the nutrient composition and labelling practices of dry or instant cereals for older infants and young children across seven Southeast Asian countries
Source: Matern Child Nutr. 2023 Dec 13;19(Suppl 2):e13603. doi: 10.1111/mcn.13603 (PMC10719057; doi:10.1111/mcn.13603)
Supplement: Supplementary file 2 — Supporting information. [file MCN-19-e13603-s001.docx]

| **Supplemental Table 2. Lists of fortificants identified in ingredient lists of commercially produced complementary foods** | |
| --- | --- |
| **Nutrient** | **Ingredients identified on labels** |
| **Vitamin A** | - vitamin A - vitamin A palmitate - retinyl acetate - vitamin A acetate - carotenoid - B-carotene |
| **Vitamin D** | - vitamin D - vitamin D3 - cholecalciferol/colecalciferol |
| **Vitamin K** | - vitamin K - vitamin K1 - phylloquinone |
| **Vitamin B1** | - vitamin B1 - thiamine hydrochloride - thiamine/thiamin - thiamine mononitrate |
| **Vitamin B2** | - vitamin B2 - riboflavin |
| **Vitamin B3** | - vitamin B3 - niacinamide - niacin - nicotinamide - niacinamide - vitamin PP |
| **Vitamin B5** | - vitamin B5 - calcium D-pantothenate - pantothenic acid - pantothenate |
| **Vitamin B6** | - vitamin B6 - pyridoxine - pyridoxine hydrochloride |
| **Vitamin B7** | - vitamin B7 |
| **Vitamin B9** | - vitamin B9 - folic acid |
| **Vitamin B12** | - vitamin B12 - cyanocobalamin |
| **Choline** | - choline - choline bitartrate |
| **Biotin** | - d-biotin - biotin |
| **Calcium** | - seaweed calcium - shell calcium - calcium - calcium phosphate - calcium lactate - calcium carbonate - tricalcium phosphate - monocalcium phosphate - dicalcium phosphate |
| **Copper** | - copper |
| **Chromium** | - crom |
| **Iodine** | - potassium iodide/iodine - iodine - kali iodide/iodine - iodide - iodized salt |
| **Iron** | - ferrous gluconate - iron - mineral iron - ferric/iron pyrophosphate - irone fumarate - ferrous sulfate - electrolytic iron - ferrous fumarate - amphoteric iron - iron fumarate - iron sulfate - ferric diphosphate - ferric orthophosphate - ferrous bisglycinate |
| **Magnesium** | - magnesium - magnesium oxide - magnesium carbonate |
| **Manganese** | - mangan |
| **Molybdenum** | - molypden |
| **Selenium** | - selenium-enriched yeast - sodium selenite - selen |
| **Zinc** | - zinc sulfate - zinc gluconate - zinc oxide - zinc |
| **DHA** | - DHA - docosahexaenoic acid (fish oil) - DHA omega-5 |
| **Vitamin/Mineral Premixes** | - compound food nutrition fortifier - vitamin mineral premix - vitamins - minerals |
| **Vitamin E** | - alpha-tocopheryl acetate - vitamin E acetate - vitamin E - mixed tocopherol - antioxidant ascorbyl palmitate and tocopherol - antioxidant tocopherol - tocopherol |
