# Supplementary material for: Benchmarking the nutrient composition and labelling practices of dry or instant cereals for older infants and young children across seven Southeast Asian countries
Source: Matern Child Nutr. 2023 Dec 13;19(Suppl 2):e13603. doi: 10.1111/mcn.13603 (PMC10719057; doi:10.1111/mcn.13603)
Supplement: Supplementary file 3 — Supporting information. [file MCN-19-e13603-s002.docx]

| **Supplementary Table 3**: Micronutrient content per 100g of fortified CPCF dry/instant cereals, median and interquartile range (IQR) by country | | | | | | | | |  |
| --- | --- | --- | --- | --- | --- | --- | --- | --- | --- |
| Country | Cambodia | Indonesia | Laos | Malaysia | Philippines | Thailand | Vietnam | All products | |
| Iron fortified (n, %) | 36 [78.3] | 86 [76.8] | 13 [46.4] | 45 [34.1] | 29 [82.9] | 10 [29.4] | 72 [74.2] | 291 [60.1] | |
| Iron content (mg) | 9.0 [32.5] | 22.0 [7.2] | 10.0 [20.6] | 10.0 [12.5] | 5.8 [13.5] | 10.0 [1.1] | 7.4 [2.5] | 10.0 [16.0] | |
| Calcium fortified (n, %) | 36 [78.3] | 88 [78.6] | 11 [39.3] | 34 [25.8] | 24 [68.6] | 9 [26.5] | 72 [74.2] | 274 [56.6] | |
| Calcium content (mg) | 429 [144] | 459 [189] | 433 [70] | 478 [86] | 432 [160] | 440 [110] | 430 [98] | 436 [122] | |
| Zinc fortified (n, %) | 36 [78.3] | 85 [75.9] | 11 [39.3] | 31 [23.5] | 22 [62.9] | 9 [26.5] | 56 [57.7] | 250 [51.7] | |
| Zinc content (mg) | 5.5 [2.1] | 4.5 [2.3] | 2.5 [4.2] | 4.5 [3.0] | 3.7 [1.8] | 2.5 [3.8] | 4.2 [1.3] | 4.3 [2.2] | |
| Vitamin B12 fortified (n, %) | 30 [65.2] | 86 [76.8] | 11 [39.3] | 30 [22.7] | 12 [34.3] | 7 [20.6] | 54 [55.7] | 230 [89.8] | |
| Vitamin B12 (mg) | 0.83 [0.50] | 1.80 [1.12] | 0.70 [0.21] | 0.80 [0.20] | 0.70 [1.40] | 0.62 [0.20] | 0.90 [0.28] | 1.03 [1.00] | |
| Vitamin A fortified (n, %) | 28 [60.9] | 88 [78.6] | 8 [28.6] | 31 [23.5] | 21 [60.0] | 9 [26.5] | 72 [74.2] | 257 [53.1] | |
| Vitamin A content (mcg) | 400 [200] | 310 [39] | 330 [30] | 350 [185] | 350 [367] | 330 [30] | 375 [150] | 330 [140] | |
| Vitamin D fortified (n, %) | 36 [78.3] | 88 [78.6] | 11 [39.3] | 31 [23.5] | 24 [68.6] | 7 [20.6] | 72 [74.2] | 274 [56.6] | |
| Vitamin D content (mcg) | 6.7 [9.6] | 10.0 [7.0] | 5.0 [5.0] | 5.0 [3.0] | 5.1 [1.0] | 5.0 [0.0] | 6.5 [2.4] | 6.5 [5.3] | |
| Copper fortified (n, %) | 0 [0.0] | 0 [0.0] | 0 [0.0] | 0 [0.0] | 0 [0.0] | 0 [0.0] | 0 [0.0] | 0 [0.0] | |
| Copper content (mg) | - | - | - | - | - | - | - | - | |
| Vitamin E fortified (n, %) | 30 [65.2] | 84 [75.0] | 11 [39.3] | 32 [24.2] | 18 [51.4] | 7 [20.6] | 66 [68.0] | 248 [51.2] | |
| Vitamin E content (mg) | 5.0 [1.0] | 5.0 [1.7] | 4.1 [1.0] | 6.0 [3.2] | 4.1 [3.4] | 5.0 [0.9] | 5.0 [1.8] | 5.0 [2.0] | |
| Vitamin K fortified (n, %) | 17 [37.0] | 22 [19.6] | 0 [0.0] | 0 [0.0] | 2 [5.7] | 0 [0.0] | 30 [30.9] | 71 [14.7] | |
| Vitamin K content (mcg) | 11.0 [1.5] | 12.5 [2.5] | - | - | 126.0 [18.0] | - | 15.7 [9.0] | 12.5 [4.7] | |
| Vitamin B1 fortified (n, %) | 39 [84.8] | 88 [78.6] | 14 [50.0] | 53 [40.2] | 29 [82.9] | 11 [32.4] | 84 [86.6] | 318 [65.7] | |
| Vitamin B1 (mg) | 0.5 [0.4] | 0.4 [0.1] | 0.5 [0.1] | 0.6 [0.2] | 0.5 [0.5] | 0.5 [0.6] | 0.7 [0.2] | 0.5 [0.3] | |
| Vitamin B2 fortified (n, %) | 27 [58.7] | 83 [74.1] | 11 [39.3] | 31 [23.5] | 18 [51.4] | 9 [26.5] | 54 [55.7] | 233 [48.1] | |
| Vitamin B2 (mg) | 0.6 [0.1] | 0.4 [0.2] | 0.4 [0.3] | 0.6 [0.3] | 0.4 [0.1] | 0.4 [0.0] | 0.6 [0.2] | 0.5 [0.3] | |
| Vitamin B6 fortified (n, %) | 30 [65.2] | 81 [72.3] | 11 [39.3] | 31 [23.5] | 18 [51.4] | 7 [20.6] | 54 [55.7] | 232 [47.9] | |
| Vitamin B6 (mg) | 0.5 [0.3] | 0.5 [0.2] | 5.0 [0.2] | 0.6 [0.4] | 0.5 [0.1] | 0.3 [0.2] | 0.8 [1.3] | 0.5 [0.3] | |
| Vitamin B3 fortified (n, %) | 30 [65.2] | 84 [75.0] | 12 [42.9] | 33 [25.0] | 20 [57.1] | 3 [8.8] | 50 [51.5] | 232 [47.9] | |
| Vitamin B3 (mg) | 6.3 [1.2] | 5.2 [1.2] | 5.0 [1.8] | 6.2 [2.0] | 4.2 [2.0] | 4.2 [0.1] | 6.7 [3.8] | 6.0 [1.9] | |
| Folic acid fortified (n, %) | 27 [58.7] | 84 [75.0] | 11 [39.3] | 30 [22.7] | 17 [48.6] | 3 [8.8] | 54 [55.7] | 226 [46.7] | |
| Folic acid content (mg) | 36.0 [53.5] | 12.0 [8.0] | 26.0 [26.5] | 30.0 [20.3] | 48.0 [13.0] | 43.0 [19.4] | 90.0 [28.0] | 26.0 [69.5] | |
| Vitamin C fortified (n, %) | 30 [65.2] | 84 [75.0] | 11 [39.3] | 34 [25.8] | 23 [65.7] | 9 [26.5] | 68 [70.1] | 259 [53.5] | |
| Vitamin C content (mg) | 61.5 [7.0] | 60.0 [21.5] | 65.0 [6.0] | 58.0 [30.0] | 35.7 [41.0] | 65.0 [5.0] | 52.0 [20.0] | 58.0 [22.6] | |
| Iodine fortified (n, %) | 20 [43.5] | 81 [72.3] | 7 [25.0] | 16 [12.1] | 15 [42.9] | 8 [23.5] | 55 [56.7] | 202 [41.7] | |
| Iodine content (mcg) | 56.3 [24.0] | 56.3 [27.0] | 47 [7.0] | 50.0 [9.5] | 47.0 [46.0] | 47.0 [7.3] | 59.0 [28.0] | 56.3 [28.0] | |
|  | | | | | | | | |  |
